# Sex‐Differences in Infrapatellar Fat Pad Size in Runners After Knee Surgery and Associations With Patellofemoral‐Related Symptoms: TRAIL Cohort Cross‐Sectional Study

**DOI:** 10.1002/jor.70300

**Published:** 2026-10-16

**Authors:** Harvi F. Hart, Kay M. Crossley, Benjamin F. Mentiplay, Elizabeth Sylvestre, Melissa J. Haberfield, Michael Girdwood, Danilo De Oliveira Silva

**Affiliations:** ^1^ Department of Kinesiology, College of Education Michigan State University East Lansing Michigan USA; ^2^ La Trobe Sport and Exercise Medicine Research Centre, School of Allied Health, Human Services, and Sport La Trobe University Bundoora Victoria Australia; ^3^ School of Physical Therapy, Faculty of Health Sciences Western University London Ontario Canada; ^4^ Graduate Advanced Health Care Practice Program, School of Physical Therapy, Faculty of Health Sciences Western University London Ontario Canada

**Keywords:** anterior cruciate ligament, Hoffa fat pad, meniscus, rehabilitation, running

## Abstract

The study aimed to examine the association between knee surgery and infrapatellar fat pad volume in female and male runners, and the association between fat pad volume and patellofemoral‐related symptoms among runners with knee surgery. This study presents a secondary exploratory analysis of a prospective cohort study. Data from female and male runners with (*n* = 104, 49% female) or without (*n* = 99, 52% female) knee surgery were included. Fat pad volumes were measured from knee magnetic resonance images. Patellofemoral‐related symptoms were evaluated using the patellofemoral pain and osteoarthritis subscale of the Knee injury and Osteoarthritis Outcome Score. Linear regression models were used to assess the associations between prior knee surgery and fat pad volume in females and males, and zero‐inflated beta regression models were used to evaluate the associations between fat pad volume and patellofemoral‐related symptoms in the knee surgery group. All models were adjusted for age, body mass, and height. Females with prior knee surgery had a smaller fat pad volume (mean difference [95% CI]: −1.80 cm^3^ [−3.02, −0.57], *p* = 0.004) than controls. However, this was not observed in males (0.30 cm^3^ [−1.39, 2.00], *p* = 0.723). Among runners with prior knee surgery, fat pad volume was not associated with symptoms in either females (average marginal effects [95% CI]: −0.035 [−0.831, 83.10], *p* = 0.931) or males (0.255 [−0.233, 23.30], *p* = 0.306). Females, but not males, exhibit a smaller fat pad volume after knee surgery compared with no surgery controls. Among runners with a history of knee surgery, fat pad volume does not appear to be associated with patellofemoral‐related symptoms.

## 1 Introduction

Running is a popular physical activity [1] that offers many health benefits, from improving musculoskeletal and cardiovascular health to boosting body composition and enhancing psychological well‐being [2, 3, 4, 5]. However, it can also be associated with lower‐extremity injuries, particularly around the knee [6, 7, 8, 9, 10]. Many individuals seek to resume running after knee surgery. However, a history of traumatic knee injury and surgery is associated with an increased risk of post‐traumatic knee osteoarthritis and persistent symptoms [11, 12, 13]. While knee injuries requiring surgery, such as anterior cruciate ligament (ACL) injuries, often occur during sports or recreational activities involving pivoting, cutting, or landing maneuvers rather than during running itself, many individuals may participate in running after recovery from knee surgery. Consequently, runners with a history of knee surgery represent an important population in whom identifying knee joint characteristics that may be relevant to joint health may improve our understanding of factors associated with symptoms following knee surgery.

Among the knee joint characteristics that might be relevant to knee joint health, the infrapatellar fat pad (hereafter, fat pad) has received increasing attention. The fat pad is a highly vascularized and innervated adipose tissue located underneath the patellar tendon within the knee joint [14, 15, 16]. It is a source of various cytokines, interleukins, adipokines, and growth factors, which are thought to influence articular cartilage and pain [17, 18, 19, 20, 21, 22]. Because of its biological activity and its potential to be disrupted during knee surgery, the fat pad size may be relevant to symptoms and joint health following knee surgery. However, its clinical relevance remains unclear. For instance, larger fat pad volume has been associated with patellar tendinopathy and patellofemoral osteoarthritis [23, 24], whereas findings in individuals following ACL reconstruction and those with knee osteoarthritis remain inconsistent [25, 26, 27, 28]. Likewise, the associations between fat pad size and knee pain present inconsistencies across studies [23, 25, 28, 29]. Furthermore, because the fat pad may be affected by surgical procedures, comparing runners with and without a history of knee surgery may provide further insight into whether differences in fat pad volume are evident within a physically active population.

Given the anatomical proximity of the fat pad to the patella and the patellofemoral compartment, fat pad volume may be related to patellofemoral symptoms. If knee surgery is associated with differences in fat pad volume, understanding whether fat pad volume is associated with patellofemoral‐related symptoms among runners with prior knee surgery may help establish the clinical relevance of such differences. However, the association between fat pad volume and patellofemoral‐related symptoms in runners with a history of knee surgery has not been investigated. Furthermore, sex differences exist in patient‐reported outcomes following ACL reconstruction [30], and in line with the Sex and Gender Equity in Research guidelines [31], it is important to examine these associations separately in females and males. A recent study found fat pad preservation technique for ACL reconstruction is associated with less anterior knee pain at 6 months and fewer chondral lesions at 1 year compared with the non‐preservation technique [32]. Therefore, we aimed to examine the association between prior knee surgery status and fat pad volume in female and male runners, and the association between fat pad volume and patellofemoral‐related symptoms among those with prior knee surgery. We hypothesized that a history of knee surgery would be associated with smaller fat pad volume in female and male runners, and that smaller fat pad volume would be associated with worse patellofemoral‐related symptoms among runners with prior knee surgery.

## 2 Methods

### 2.1 Study Design and Participants

This exploratory cross‐sectional analysis used baseline data from the trajectory of knee health in runners (TRAIL) study—a prospective cohort study of runners with (surgery group) and without (control group) a history of knee surgery [33, 34]. The full methodological details are published elsewhere [33, 34]. Briefly, the inclusion criteria for all participants at enrollment were: (i) aged 18 to 50 years and (ii) running ≥ 3 times and ≥ 10 km per week on average in the past 6 months. Individuals were included in the surgery group if they had anterior or posterior cruciate ligament, meniscal, chondroplasty, collateral ligament, or arthroscopy surgery. Individuals with a history of intra‐articular knee fracture, arthroplasty, osteotomy, patellar tenotomy, lateral retinacular release, or other lower limb surgery were excluded. Participants were included in the control group if they had no history of lower limb surgery throughout their lifetime. Individuals were not included if they had any musculoskeletal traumatic or overuse injury in the previous 6 months that required a period of non‐weight‐bearing for ≥ 24 h. Ethical approval was obtained from La Trobe University (HEC‐19524) Human Research Ethics Committee. All participants provided written informed consent.

### 2.2 Fat Pad Volumetric Assessment

Fat pad volumes were assessed in participants in the surgery and control groups using knee magnetic resonance imaging (MRI) acquired at baseline using a 3 T scanner (Signa Pioneer, General Electric Healthcare, Milwaukee, USA) and an 18‐channel knee coil. For participants with a history of multiple unilateral or bilateral knee surgeries, the index knee was selected based on the reference surgery, defined as the most significant procedure (ACL reconstruction > meniscus and/or cartilage surgery > other). In individuals with bilateral knee surgery of the same type, the most recently operated knee was selected as the index knee. For the control group, the index knee was randomly selected before data collection [33, 34]. Imaging sequences included proton density‐weighted fat‐suppressed fast spin‐echo sequences in the sagittal, axial, and coronal planes; a sagittal T2 mapping multi‐echo spin‐echo sequence; and a sagittal fast spoiled gradient echo sequence. Four raters were randomly assigned 205 available knee MRI scans to measure fat pad volumes using sagittal T2 mapping multi‐echo spin‐echo sequences. Fat pad segmentations were completed using open‐source software (3D Slicer software Version 5.5.0, Surgical Planning Laboratory at Brigham and Women's Hospital and the MIT Artificial Intelligence Laboratory, Massachusetts). The fat pad was segmented by manually tracing the borders of the fat pad (Figure 1) from the first to the last slice, where the structure was visible. Once all slices were digitized for each participant, fat pad volume was computed. The interrater reliability of fat pad volume segmentation was verified using 11 knee MRI scans from the same dataset (Intraclass correlation coefficient [95% confidence interval]: 0.982 [0.956, 0.995]) before commencing the segmentation of all MRIs for this study.

**Figure 1:**
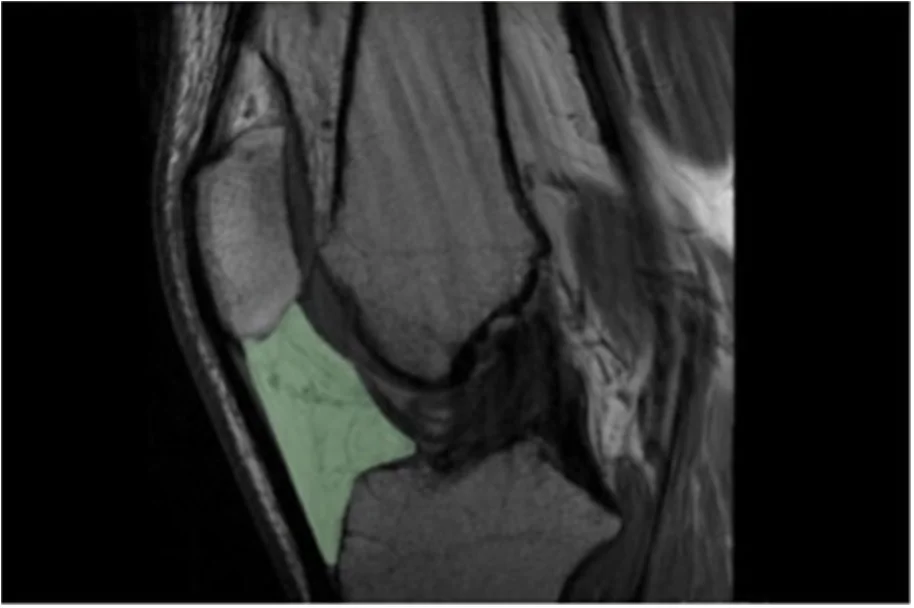
Sagittal T2‐mapped multi‐echo spin‐echo knee magnetic resonance imaging showing segmented infrapatellar fat pad.

### 2.3 Patellofemoral‐Related Symptoms

All participants completed the Knee injury and Osteoarthritis Outcome Score for the patellofemoral pain and osteoarthritis (KOOS‐PF), a patellofemoral‐specific subscale developed separately from the original KOOS [35]. A normalized score from 0 to 100 is calculated, where a score of zero represents maximum patellofemoral‐related symptoms and functional limitations.

### 2.4 Statistical Analyses

As this was a secondary exploratory analysis, no a priori sample size calculation was performed; the sample size was determined based on the available participants within the existing dataset. Participant characteristics are presented as means with standard deviation. Descriptive characteristics were summarized separately for females and males with a history of knee surgery and their respective controls. Continuous variables are presented as means with standard deviation, while categorical variables are presented as *n* (%). To evaluate the associations between prior knee surgery and fat pad volumes in runners, separate linear regression models for females and males were conducted. After testing the assumptions for linear regression models, linear regression models with robust standard errors were used for mild violations of the underlying assumptions. The models were adjusted for the influence of age, body mass, and height (Stata/SE 17.0, StataCorp, College Station, TX, USA). Regression coefficients (β) with 95% confidence intervals (CIs) were reported, where β represents the adjusted mean difference in fat pad volume between runners with and without a history of knee surgery.

To evaluate the associations between fat pad volume and patellofemoral‐related symptoms among females and males with prior knee surgery, zero‐inflated beta regression models were used. In this cohort, KOOS‐PF scores were often high (median 95.5), resulting in a skewed distribution. Because traditional linear regression assumes unbounded and normally distributed outcomes, it was considered inappropriate for these data. To address this, the original 0 to 100 KOOS‐PF scores (where lower values represent greater symptom severity) were rescaled to the 0‐1 interval for beta regression, which accommodates outcomes bounded between 0 and 1. As the glmmTMB package used only supports zero‐inflated beta regressions, scores therefore needed to be reversed so that “perfect” original scores (e.g., KOOS‐PF = 100) were transformed to 0, allowing proper modeling of zero‐inflation. Diagnostics for Hierarchical/Regression Models (DHARMa) were used to evaluate model fit. Analyses were performed in R using the glmmTMB, DHARMa, ggeffects, and marginaleffects packages (RStudio Version 2026.01.2 + 418, Posit Team 2025). All models were adjusted for age, body mass, and height, and the associations were evaluated separately for females and males. In males, initial beta regression models failed to meet model assumptions based on DHARMa residual diagnostics. Specifically, the residuals‐versus‐predicted plot showed significant quantile deviations, and the combined adjusted quantile test was also significant, indicating model misfit. To address potential nonlinearity, fat pad volume was first modeled using natural cubic splines (df = 3), while age, body mass, and height were retained as linear covariates. However, residual diagnostics continued to indicate significant deviations, suggesting that additional sources of nonlinearity were not adequately captured. Consequently, nonlinear effects for covariates were explored by introducing natural cubic splines for age, height, and body mass in various combinations. Knot locations were determined automatically using the default quantile‐based approach implemented in the ns() function in the R splines package. Models including nonlinear terms for individual covariates did not sufficiently improve model fit. The final model included nonlinear effects for age and height, as it satisfied the model assumptions based on DHARMa residual diagnostics, and yielded the lowest AIC and BIC, indicating the best balance between model fit and parsimony. Results from zero‐inflated beta‐regressions were presented as average marginal effects and 95% confidence intervals, accounting for the zero‐inflated portion of the model (marginaleffects package). The average marginal effects and 95% confidence intervals were then back‐transformed to the original scale.

## 3 Results

Data from 104 participants in the knee surgery group and 99 in the control group were included in the analysis (Table 1). Overall, KOOS‐PF scores were generally high among participants with a history of knee surgery, suggesting low levels of patellofemoral‐related symptoms. The proportion of participants achieving the maximum KOOS‐PF score was 25.5% among females and 42.3% among males with a history of knee surgery.

**Table 1:** Participants' characteristics.

	Knee surgery (*n* = 104)		Control (*n* = 99)	
	Females (*n* = 51)	Males (*n* = 53)	Females (*n* = 52)	Males (*n* = 47)
Age, years	34.2 ± 7.6	35.6 ± 7.3	32.3 ± 6.7	34.4 ± 7.2
Body mass, kg	64.2 ± 9.7	79.3 ± 8.3	62.6 ± 7.9	77.0 ± 10.7
Height, m	1.69 ± 0.06	1.81 ± 0.06	1.67 ± 0.05	1.82 ± 0.07
Fat pad volume, cm^3^	21.17 ± 4.13	29.93 ± 5.09	22.41 ± 3.46	29.92 ± 4.32
KOOS‐PF	87 ± 13	93 ± 11	96 ± 10	96 ± 8
Time post‐surgery, years	8.6 ± 7.1	10.7 ± 8.1		
Surgery type, *n* (%)				
* ACLR*	36 (70.59)	25 (47.17)		
* Meniscus*	9 (17.65)	26 (49.06)		
* Other*	6 (11.76)	2 (3.77)		

*Note:* Data are presented as mean ± standard deviation, unless otherwise stated. KOOS‐PF is the patellofemoral pain and osteoarthritis subscale of the Knee injury and Osteoarthritis Outcome Score. Fat pad is the infrapatellar fat pad, and ACLR is anterior cruciate ligament reconstruction.

Among female runners, a history of knee surgery was associated with a smaller fat pad volume than in controls (β coefficient [95% CI]: −1.80 cm^3^ [−3.02, −0.57], *p* = 0.004). However, these associations were not found in males (0.30 cm^3^ [−1.39, 2.00], *p*= 0.723) (Figure 2). Complete regression model results are presented in Supplementary Table 1.

**Figure 2:**
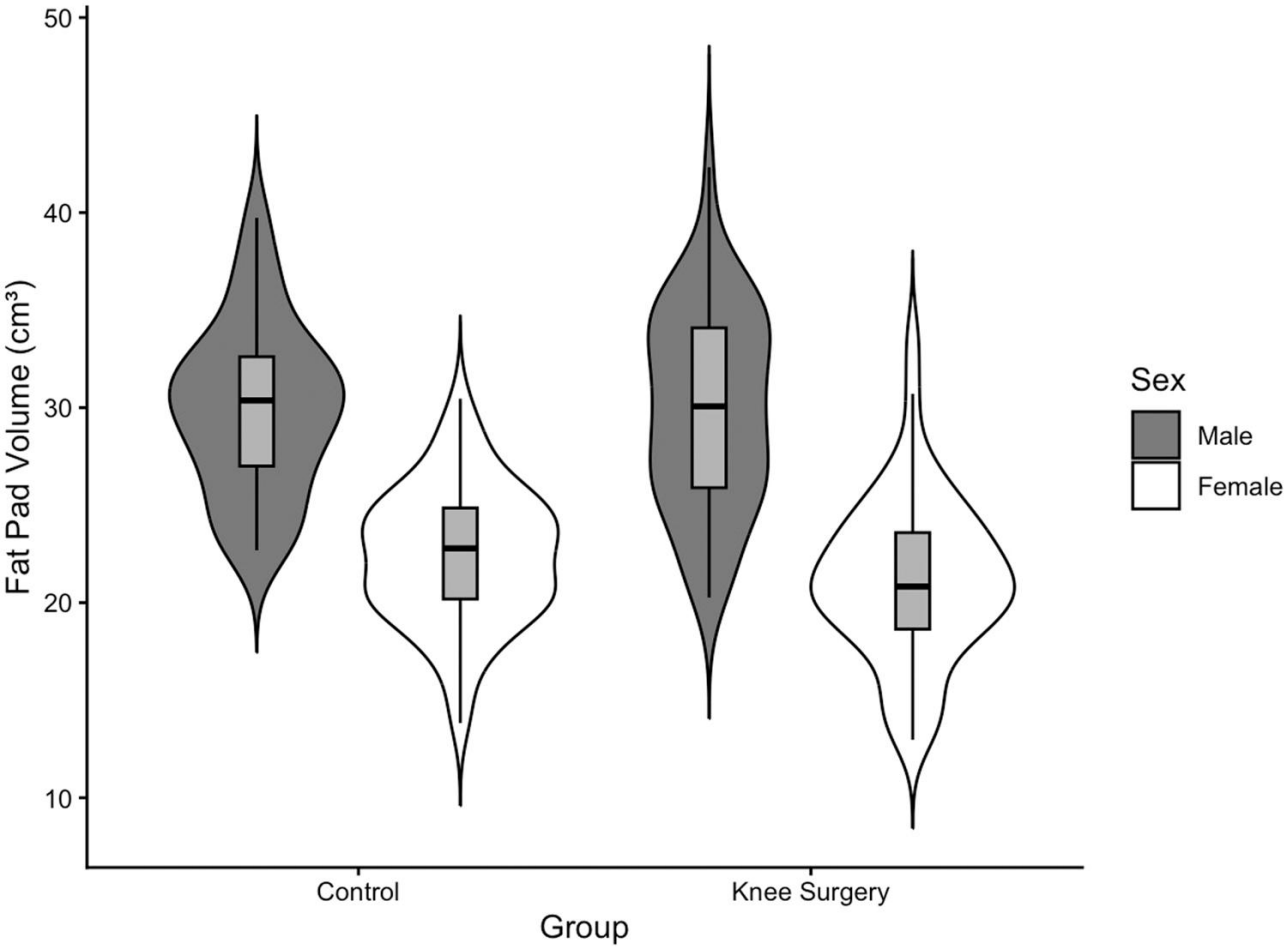
Violin plots illustrating fat pad volume (cm^3^) across groups stratified by sex. The width of each violin reflects the distribution density, with wider sections indicating a higher concentration of observations. Embedded boxplots display the median and interquartile range.

There were no associations observed between fat pad volume and KOOS‐PF scores in females and males in the knee surgery group (Figure 3). Among female runners, the predicted KOOS‐PF scores ranged from 88 to 87 across fat volumes of 12–34 cm^3^ (*average marginal effects* [95% CI]: −0.035 [−0.831, 83.10], *p* = 0.931). Among male runners, the predicted KOOS‐PF scores ranged from 95 to 98 across fat volumes of 20–42 cm^3^ (0.255 [−0.233, 23.30], *p* = 0.306). Complete regression model results are presented in Supplementary Table 2.

**Figure 3:**
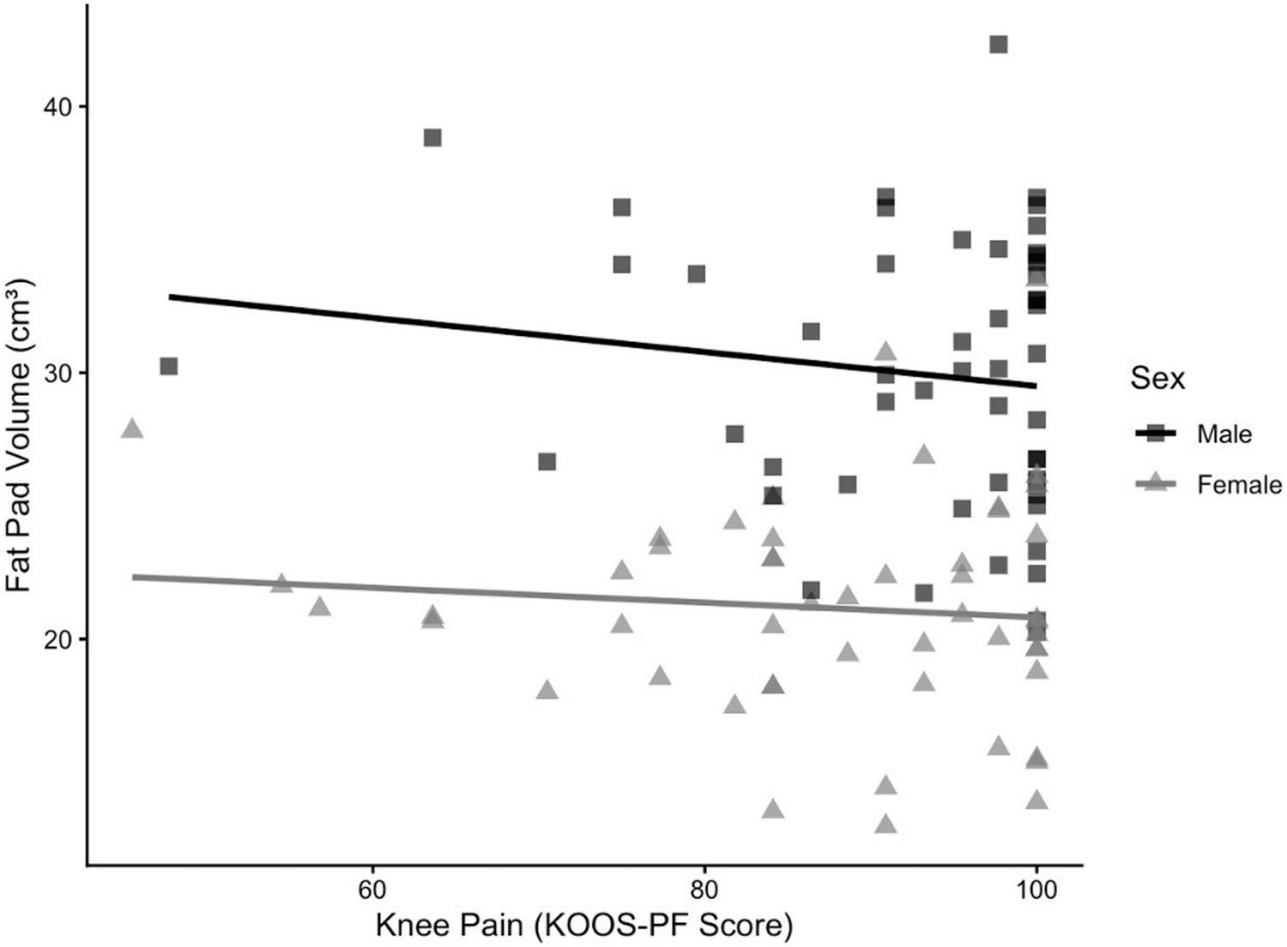
Scatter plot of knee pain versus fat pad volume (cm^3^) among runners with knee surgery, stratified by sex. KOOS‐PF is the Patellofemoral Pain and Osteoarthritis Subscale of the Knee injury and Osteoarthritis Outcome Score. A higher score on the KOOS‐PF indicates less knee pain.

## 4 Discussion

Among runners, females with a history of knee surgery, but not males, appear to have a less voluminous fat pad compared to controls; however, fat pad volume did not appear to be associated with patellofemoral‐related symptoms in either females or males with knee surgery.

Our results are somewhat consistent with our hypothesis, showing smaller fat pad volume in females with a history of knee surgery compared to controls; however, this was not observed in males. Prior research, using a combined sample of females and males, found no differences in fat pad volume between individuals 1‐year post‐ACL reconstruction and uninjured controls [25]. The average fat pad volumes in our surgery group were 21 cm^3^ in females and 30 cm^3^ in males, both smaller than the average volume (35 cm^3^) observed in younger individuals 1‐year after ACL reconstruction [25]. The smaller fat pad volumes observed in our study, compared with those reported in individuals 1 year after ACL reconstruction [25], may reflect the substantially longer time since surgery in our cohort (~10 years). It may be that fat pad volume change is more pronounced during the early postoperative period and gradually attenuates over time. However, differences in study populations may also contribute to these findings, as our cohort consisted of runners, whereas the previous study included participants from mixed‐sport populations.

It is also important to consider the types of surgeries performed across sexes in this study when considering sex‐specific differences. For example, 71% of females and 47% of males underwent ACL reconstruction, while meniscal surgery was performed in 18% of females and 49% of males, and other knee surgeries were reported in 12% of females and 4% of males. The type of surgery may affect the fat pad structure differently, due to the specific surgical procedures involved. For example, during ACL reconstruction, the fat pad may be disrupted or shaved to improve intra‐articular visibility [36]. Our sensitivity analyses comparing females and males with ACL reconstruction to controls produced similar results (Females: −1.98 cm^3^ [95% CI: −3.44, −0.53], *p* = 0.008; Males: 0.34 cm^3^ [−2.65, 2.00], *p* = 0.772). Overall, the observed sex differences may reflect sex‐specific patterns of responses to injury, recovery, and adaptation between females and males, potentially influenced by factors such as hormones and body composition [37, 38]. Although differences in fat pad volume were observed among female runners with and without knee surgery, the magnitude of these differences was small (< 10%). Consequently, it remains unclear whether these findings are clinically meaningful and warrant further investigation.

Contrary to our hypothesis, fat pad volume did not appear to be associated with patellofemoral‐related symptoms in females and males with prior knee surgery. Earlier research has found no associations between fat pad volume and knee pain in individuals after ACL reconstruction [25]. In contrast, studies in individuals with knee osteoarthritis have reported associations between both fat pad volume and maximal cross‐sectional area with knee pain [23, 29]. In the present cohort, runners in the knee surgery group reported minimal knee pain on the KOOS‐PF. A previous publication from this cohort indicates these individuals remained regular runners after knee surgery [39]. This subgroup may represent individuals who continue running despite prior knee surgery and may have relatively favorable symptom profiles, which could partly explain the lack of associations between fat pad volume and patellofemoral‐related symptoms. Alternatively, fat pad size alone may not be associated with knee pain in individuals with knee injuries; instead, chronic alterations in fat pad composition may also be more relevant to knee symptoms. For example, the fat pad preservation technique for ACL reconstruction was associated with lower synovial inflammatory markers and reduced fat pad fibrosis at 1‐year post‐ACL reconstruction [32]. Another consideration is time post‐operatively; the lack of association between fat pad volume and knee pain may simply represent long post‐operative duration (~10 years), during which the associations may have attenuated. Longitudinal investigations are needed to better understand the sex‐specific role of fat pad volume and morphology in symptoms and degenerative changes over time following knee surgery.

From a clinical perspective, although female runners with a history of knee surgery exhibited smaller fat pad volumes than controls, fat pad volume does not appear to be associated with patellofemoral‐related symptoms in either sex in runners. As such, the present cross‐sectional findings do not provide evidence of clinical relevance of fat pad volume for the assessment of patellofemoral‐related symptoms in this population. From a research perspective, the observed sex‐differences suggest that alterations in fat pad morphology may persist long after knee surgery. Future longitudinal studies should investigate whether other fat pad characteristics, such as fibrosis, inflammatory status, or other morphological features, are associated with clinical outcomes.

In our study, we reliably assessed fat pad volume, which provides a better indicator of fat pad size than the maximal cross‐sectional area from MRI. The results of the present study should be interpreted considering several limitations. The cross‐sectional design of the study precludes us from establishing any causal relationship between fat pad size, knee pain, and knee surgery history. Consequently, there is a need for longitudinal research to elucidate alterations in fat pad size and its influence on knee pain over time among runners. The type of surgery (e.g., ACL, meniscal, chondroplasty, or arthroscopy) may influence fat pad size, but surgery type was not considered in the analyses. However, sensitivity analyses limited to those with a history of ACL reconstruction surgery yielded similar findings. Further to this, runners with prior surgery reported minimal symptom severity, which may have limited our ability to detect associations between fat pad volume and patellofemoral‐related symptoms. However, we used zero‐inflated beta regression models to account for the bounded and highly skewed distribution of the scores. Further research is needed to determine whether the relationship between fat pad volume and patellofemoral‐related symptoms differs among runners with previous knee surgery across varying levels of knee symptoms severity. Another consideration is that the relatively small sample size for each sex may have limited the precision and stability of the estimates from the association analyses. Thus, these findings should be interpreted cautiously. The results of this study are most generalizable to runners with or without a history of knee surgery who remain physically active. However, external validity may be limited to other populations, including non‐running individuals, less active individuals, or those with more severe knee symptoms. Future studies should address the methodological limitations of the present study and use longitudinal designs to determine whether fat pad size and morphology can be used to guide clinical assessment and management of symptoms and longer‐term outcomes in runners following knee surgery.

## 5 Conclusion

Females, but not males, with prior knee surgery have a smaller fat pad volume compared with their uninjured counterparts. Fat pad volume does not appear to be associated with patellofemoral‐related symptoms in female or male runners with prior knee surgery.

## Declaration of Generative AI and AI‐Assisted Technologies in the Writing Process

During the preparation of this work, generative AI Technology (Grammarly AI) was used to improve the clarity and readability of several sentences. After using this tool/service, the content was reviewed and edited as needed for accuracy.

## Author Contributions

Conceptualization: Harvi Hart, Danilo De Oliveira Silva; Investigation: Harvi Hart, Elizabeth Sylvestre; Methodology: Harvi Hart, Danilo De Oliveira Silva; Formal analysis: Harvi Hart, Benjamin Mentiplay, Michael Girdwood; Data curation: Harvi Hart, Benjamin Mentiplay, Michael Girdwood; Visualization: Harvi Hart; Writing – original draft preparation: Harvi Hart; Writing – review and editing: Kay Crossley, Benjamin Mentiplay, Elizabeth Sylvestre, Melissa Haberfield, Michael Girdwood, Danilo De Oliveira Silva; Funding acquisition: Kay Crossley, Benjamin Mentiplay, Melissa Haberfield, Michael Girdwood, Danilo De Oliveira Silva. All authors have read and agreed to the submitted version of the manuscript.

## Conflicts of Interest

The authors declare no conflicts of interest.

## Supporting information


Supporting File 1

Supporting File 2

## Data Availability

The data that support the findings of this study are available on request from the corresponding author. The data are not publicly available due to privacy or ethical restrictions. Data are available upon reasonable request.
