# Supplementary material for: Sex‐Differences in Infrapatellar Fat Pad Size in Runners After Knee Surgery and Associations With Patellofemoral‐Related Symptoms: TRAIL Cohort Cross‐Sectional Study
Source: J Orthop Res. 2026 Oct 16;44(10):e70300. doi: 10.1002/jor.70300 (PMC13673570; doi:10.1002/jor.70300)
Supplement: Supplementary file 1 [file JOR-44-0-s001.docx]

**Supplementary Table 2:** Conditional model coefficients from zero-inflated beta regression analyses examining associations between fat pad volume and KOOS-PF scores among female and male runners with a history of knee surgery.

|  | **Estimate** | **95% CI** | **p-value** |
| --- | --- | --- | --- |
| **Females** |  |  |  |
| Fat pad volume, cm^3^ | 0.003 | -0.073, 0.080 | 0.931 |
| Body mass, kg | 0.03 | -0.001, 0.06 | 0.059 |
| Age, years, | 0.01 | -0.02, 0.05 | 0.494 |
| Height, cm | -0.04 | -0.09, 0.02 | 0.202 |
| **Males** |  |  |  |
| Fat pad volume, cm^3^ (spine 1) | -0.64 | -2.39, 1.11 | 0.474 |
| Fat pad volume, cm^3^ (spine 2) | -0.14 | -3.37, 3.09 | 0.933 |
| Fat pad volume, cm^3^ (spine 3) | -1.28 | -2.93, 0.37 | 0.128 |
| Body mass, kg | 0.04 | 0.01, 0.07 | 0.021 |
| Age, years (spine 1) | 0.70 | -0.58, 1.97 | 0.286 |
| Age, years (spine 2) | 0.85 | -1.37, 3.07 | 0.452 |
| Age, years (spine 3) | -0.35 | -1.59, 0.88 | 0.575 |
| Height, cm (spine 1) | -1.57 | -3.05, -0.09 | 0.038 |
| Height, cm (spine 2) | -0.85 | -6.13, 4.43 | 0.752 |
| Height, cm (spine 3) | 2.89 | 0.72, 5.06 | 0.009 |

*Note.* Male models included natural cubic spline terms for fat pad volume, age, and height. KOOS-PF is the Knee injury and Osteoarthritis Outcome Score for patellofemoral pain and osteoarthritis
