# Supplementary material for: Sex‐Differences in Infrapatellar Fat Pad Size in Runners After Knee Surgery and Associations With Patellofemoral‐Related Symptoms: TRAIL Cohort Cross‐Sectional Study
Source: J Orthop Res. 2026 Oct 16;44(10):e70300. doi: 10.1002/jor.70300 (PMC13673570; doi:10.1002/jor.70300)
Supplement: Supplementary file 2 [file JOR-44-0-s002.docx]

Supplementary Table 1: Linear regression models examining the association between a history of knee surgery and infrapatellar fat pad volume in female and male runners

|  | β (cm³) | 95% CI | p-value | R^2^ |
| --- | --- | --- | --- | --- |
| Females |  |  |  |  |
| History of knee surgery | -1.80 | -3.02, -0.57 | 0.004 | 0.305 |
| Age, years | 0.08 | -0.03, 0.18 | 0.149 |  |
| Height, cm | 0.24 | 0.12,0.36 | <0.001 |  |
| Body mass, kg | 0.10 | 0.03, 0.18 | 0.007 |  |
| Males |  |  |  |  |
| History of knee surgery | 0.30 | -1.39, 2.00 | 0.723 | 0.199 |
| Age, years | -0.04 | -0.15, 0.07 | 0.488 |  |
| Height, cm | 0.29 | 0.13, 0.45 | 0.001 |  |
| Body mass, kg | 0.04 | -0.06, 0.15 | 0.409 |  |
